# Predictive Model of Recovery to Prefracture Activities-of-Daily-Living Status One Year after Fragility Hip Fracture

**DOI:** 10.3390/medicina60040615

**Published:** 2024-04-09

**Authors:** Nitchanant Kitcharanant, Pichitchai Atthakomol, Jiraporn Khorana, Phichayut Phinyo, Aasis Unnanuntana

**Affiliations:** 1 Department of Orthopaedics, Faculty of Medicine, Chiang Mai University, Chiang Mai 50200, Thailand; nk_win@hotmail.com; 2 Division of Pediatric Surgery, Department of Surgery, Faculty of Medicine, Chiang Mai University, Chiang Mai 50200, Thailand; nanji22@gmail.com; 3 Center for Clinical Epidemiology and Clinical Statistics, Faculty of Medicine, Chiang Mai University, Chiang Mai 50200, Thailand; phichayutphinyo@gmail.com; 4 Department of Family Medicine, Faculty of Medicine, Chiang Mai University, Chiang Mai 50200, Thailand; 5 Musculoskeletal Science and Translational Research (MSTR), Faculty of Medicine, Chiang Mai University, Chiang Mai 50200, Thailand; 6 Department of Orthopaedic Surgery, Faculty of Medicine Siriraj Hospital, Mahidol University, 2 Wanglang Road, Bangkok 10700, Thailand; uaasis@gmail.com

**Keywords:** fragility hip fracture, functional recovery, predictive model

## Abstract

*Background and Objectives*: Achieving prefracture functional status is a critical objective following a hip fracture, yet fewer than half of patients reach this milestone. The adoption of tools for assessing functional outcomes is increasingly recognized as essential for evaluating recovery following treatment for fragility hip fractures. We developed multivariable clinical prediction criteria to estimate the likelihood of patients regaining their prefracture activities-of-daily-living (ADL) status one year after sustaining a fragility hip fracture. *Materials and Methods*: A retrospective cohort of patients treated for fragility hip fractures at a university-affiliated tertiary care center between February 2017 and April 2019 served as the basis for developing and internally validating the clinical prediction criteria. We applied a multivariable fractional polynomial method to integrate several continuous predictors into a binary logistic regression model. *Results*: The study included 421 patients, 324 (77%) of whom reported regaining their prefracture activities-of-daily-living level one year after experiencing fragility hip fractures. Significant predictors, such as the prefracture Barthel index, EQ-VAS score, and treatment modality, were incorporated into the predictive model. The model demonstrated excellent discriminative power (AuROC of 0.86 [95% CI 0.82–0.91]) and satisfactory calibration. *Conclusions*: The predictive model has significant discriminative ability with good calibration and provides clinicians with a means to forecast the recovery trajectories of individual patients one year after a fragility hip fracture, which could be useful because prompt clinical decision-making is aided by this information. Patients and caregivers can also be counseled and encouraged to follow up with the medical activities and interventions deemed essential by doctors who used the prediction tool. Access to the model is provided through a web application. External validation is warranted in order to prove its applicability and generalizability.

## 1. Introduction

Bone micro-architecture degradation and decreased bone mineral density are the hallmarks of osteoporosis [1]. Osteoporosis and its most catastrophic outcome, a fragility hip fracture, are becoming increasingly common as more populations become older worldwide [2] A simple fall is typically the cause of a fragility hip fracture, which increases morbidity, death, and disability [3]. Within the first year, elderly individuals with hip fractures caused by falls had a 25% mortality risk [4]. Fragility hip-fracture patients are expected to experience greater morbidity and mortality compared to non-fracture patients in the same age group; in fact, their death rate is eight times higher than that of older adults in the general population [5]. In addition, less than 50% of patients who sustain a fragility hip fracture are able to resume their pre-injury level of ambulation, and about 20% or so are immobile at one year following the injury [6]. In the two years after a fragility hip fracture, 20% of individuals will experience a subsequent fragility fracture. If the patient suffers another fragility fracture, the functional outcome can worsen [7].

By 2050, the global incidence of hip fractures is projected to increase to approximately 4.5 million cases annually, predominantly due to the aging of the population [8]. The Asia-Pacific region is expected to bear nearly half of this burden [9]. In Thailand, there has been a notable increase in the incidence of hip fracture, from 151.2 per 100,000 individuals in 1997 to 181.0 per 100,000 individuals in 2006. Projections indicate a surge to 436.1 per 100,000 by 2050 [10]. The necessity of surgical intervention in most hip-fracture cases underscores the potential socioeconomic impact that healthcare systems could face if these issues are not adequately addressed. Additionally, the decline in physical function, primarily due to diminished mobility, often results in increased dependency, necessitating caregiver assistance for activities of daily living and thereby increasing the recovery burden on families and caregivers [11].

Regaining prefracture functional status presents a significant challenge, with fewer than half of the patients achieving this goal [12]. The recovery timeframe for individual activities of daily living (ADL) ranges from 4 to 11 months [13], suggesting that the optimal period for intervention, such as comprehensive rehabilitation, is within the first year following the fracture. Early initiation of postfracture rehabilitation not only enhances ambulation and ADL performance but also contributes to a reduction in the mortality associated with hip fractures [14]. Conversely, delayed rehabilitation can lead to prolonged immobilization, exacerbating conditions in patients of advanced age and elevating the risk of mortality [15]. The evidence supports the efficacy of intensive rehabilitation programs in improving the ability to perform self-care and walk after hip fracture [14], underscoring the value of predicting patients’ capacity for functional recovery to facilitate early and appropriate interventions.

The evaluation of functional outcomes is increasingly recognized as an essential component of posttreatment assessments for hip fractures. However, models specifically tailored to predicting functional recovery following these injuries are scarce. Our study aimed to devise and internally validate a set of multivariable clinical prediction criteria aimed at determining the likelihood of patients regaining their preinjury ADL status one year after a fragility hip fracture. This predictive model is intended to support clinicians in advising patients and devising targeted strategies to enhance the rates of functional recovery during the posttreatment phase.

## 2. Materials and Methods

### 2.1. Design and Setting

To develop the clinical prediction criteria, we employed a retrospective observational cohort design within a prognostic prediction research framework. We analyzed a specific patient demographic, namely, patients admitted for fragility hip fractures at a university-affiliated tertiary care center between February 2017 and April 2019. The study strictly adhered to the ethical guidelines concerning human subjects research set forth by institutional and national research committees, and it was consistent with the 1964 Helsinki Declaration and its later amendments. Institutional Review Board approval was obtained (approval number 512/2022), and the study was duly registered in the Thai Clinical Trials Registry (registration number TCTR20230710001).

### 2.2. Study Patients

To identify relevant cases within electronic medical records, patient selection was guided by the International Classification of Diseases, Tenth Revision (ICD-10), specifically by codes S7200 (fracture of the femoral neck) and S7210 (intertrochanteric fracture). The study included individuals aged 50 years and older who had experienced low-energy trauma hip fractures with a follow-up period of at least one year or until death. Patients with multiple fractures or fractures attributed to malignancy, as verified by pathological examination, were excluded from the study.

### 2.3. Treatment Protocol

Within our institution, a comprehensive care approach for fragility hip-fracture patients is employed, and it involves a multidisciplinary team consisting of orthopedic surgeons, geriatricians, physical therapists, and nurses. The treatment pathway begins with a preoperative assessment by a geriatrician, which is followed by surgical intervention performed by an orthopedic surgeon as soon as the patient’s condition is optimized. For those deemed medically unfit, a conservative treatment approach is adopted.

Rehabilitation, led by physical therapists, is initiated promptly to prevent complications from extended immobility. Patients are provided with individualized home exercise plans, and caregivers receive guidance on ongoing care. The treatment and management of osteoporosis are guided by specialists in metabolic bone disease [16]. Educational sessions on osteoporosis and fall prevention are conducted for both patients and caregivers [11]. After discharge, patients are monitored through telephone follow-ups at 3-month intervals, which later transition to annual check-ins.

### 2.4. Data Collection

The collected data included demographic and clinical variables such as age, sex, body mass index, Charlson Comorbidity Index (CCI) score, prefracture ambulatory status (categorized on a scale from bedridden to outdoor independent), prefracture Barthel Index (BI), prefracture EQ-VAS score, fracture type (femoral neck or intertrochanteric), and treatment modality (ranging from conservative to arthroplasty).

The BI evaluates a patient’s ability to perform 10 basic activities of daily living and their mobility, with a scoring system that assigns points to each activity. The total score ranges from 0 (total dependence) to 100 (independence), with intermediate categories indicating varying levels of dependence [17]. The BI’s validity extends to assessing postoperative functionality following hemiarthroplasty [18] and conducting assessments via telephone [19]. ADL recovery was gauged through direct telephone interviews one year after the fracture, comparing BI scores from before the fracture to scores at the one-year follow-up.

The EQ-VAS is a self-administered visual analog scale in which patients rate their current health status from 0 (worst possible health) to 100 (best possible health) [20]. This assessment provides insight into patients’ perceptions of their health status.

### 2.5. Statistical Analysis

The data distribution was assessed using histograms and the Shapiro–Wilk test. Continuous variables following a normal distribution were summarized as means ± standard deviations, whereas nonnormally distributed data were described using medians and interquartile ranges. Categorical variables are reported as counts and percentages, and Fisher’s exact test was used for the comparative analysis.

Five candidate predictors were identified for inclusion in our analysis: age, CCI score, treatment modality, prefracture BI score, and prefracture EQ-VAS score. These criteria were selected due to their recognized significance in the literature as predictors of functional recovery after a fragility hip fracture [21]. We utilized multivariable fractional polynomial (MFP) algorithms to integrate multiple continuous predictors into a binary logistic regression model, thereby ensuring that the linearity assumption was not violated. This process involved a two-stage approach. The first stage was a backward elimination procedure that was used to remove nonsignificant predictors based on odds ratios, *p*-values, and clinical relevance. The second stage focused on identifying the optimal fractional polynomial transformations for each predictor and utilized a closed test algorithm.

The discriminative power of the model was evaluated by plotting the receiver operating characteristic curve, and model calibration was assessed through calibration plots. Internal validation of the model was achieved through bootstrap resampling, which was performed 1000 times to estimate model optimism and the shrinkage factor.

A web application was developed to operationalize the predictive model, enabling clinicians to estimate the odds of patients returning to their prefracture functional status one year after osteoporotic hip fracture. This application also presents predicted likelihood ratio (LHR) values with designated cutoff points, with the lowest LHR value corresponding to the greatest risk of not achieving prefracture ADL status and the highest LHR value corresponding to a strong likelihood of regaining such status. Statistical analyses were carried out by all authors using Stata Statistical Software, version 16 (StataCorp LLC, College Station, TX, USA), with a two-tailed *p*-value of < 0.05 considered to indicate statistical significance.

## 3. Results

**Figure 1:**
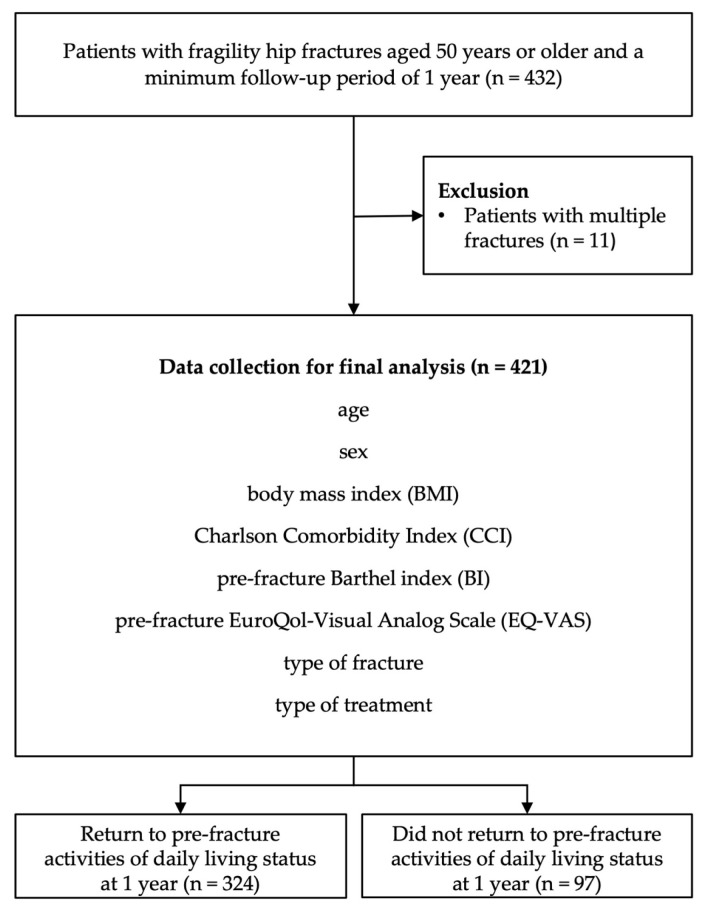
Flow diagram of patient-recruitment process.

Between February 2017 and April 2019, 485 patients were admitted for osteoporotic hip fractures. Of these, 432 met the inclusion criteria of having sustained low-energy trauma fractures, being aged 50 years or older, and having a minimum of one year of follow-up. Eleven patients were excluded due to having multiple fractures, leaving 421 patients for the final analysis (Figure 1).

Among these 421 patients, 324 (77%) successfully regained their prefracture ADL status. Patients who returned to their prefracture status were significantly younger (*p* < 0.001) and more likely to have a CCI score less than 5 (*p* < 0.001). Fewer patients in this group received conservative treatment compared to those in the group who did not regain their prefracture ADL status (*p* < 0.001). In addition, patients who regained their prefracture status had higher prefracture BI and EQ-VAS scores (both *p*-value < 0.001; Table 1).

**Figure 2:**
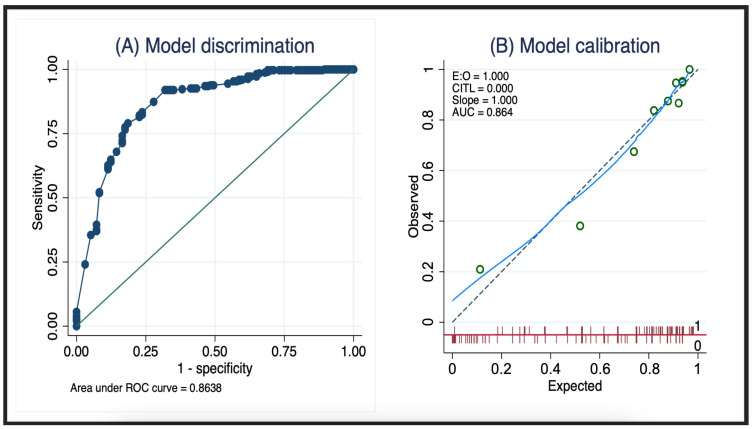
(**A**) The model discriminative power and (**B**) calibration.

For the multivariable logistic regression analysis, age, CCI score, type of treatment, prefracture BI score, and prefracture EQ-VAS score were initially considered. Following the application of MFP techniques, the prefracture BI score, EQ-VAS score, and type of treatment emerged as significant predictors and were included in the predictive model. Age and the CCI were excluded due to their nonsignificant associations with the outcome. The details of the parameter coefficients, along with their 95% confidence intervals (CIs) and *p*-values, are given in Table 2. The model demonstrated excellent discriminative power, with an area under the receiver operating characteristic curve (AuROC) of 0.86 (95% CI 0.82–0.91; Figure 2A), and showed good calibration (Figure 2B).

**Figure 3:**
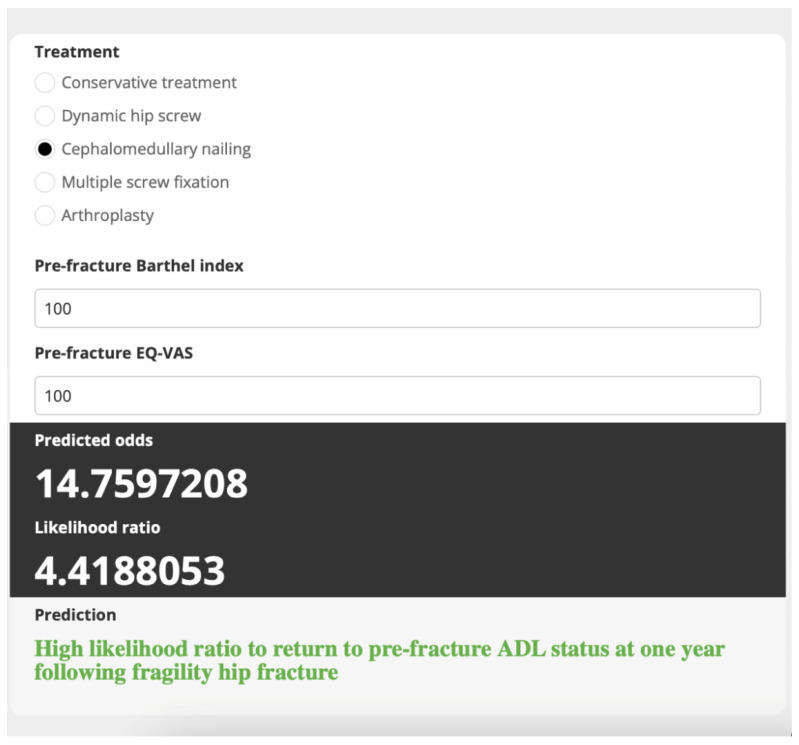
The developed web application (Scenario 1).

**Figure 4:**
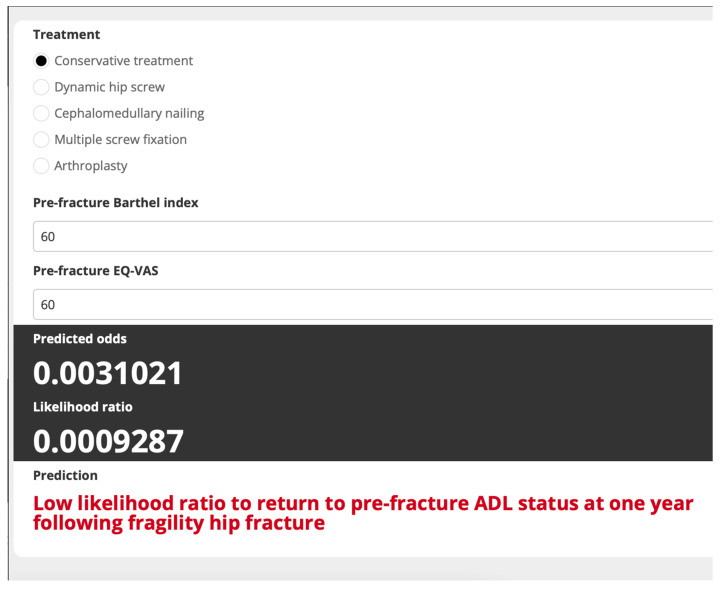
The developed web application (Scenario 2).

Internal validation via bootstrap resampling confirmed the robustness of the model, yielding a consistent AuROC of 0.86 (95% CI 0.81–0.91) and minimal optimism (0.007, range −0.083 to 0.101). The shrinkage factor was estimated to be 0.959. The developed web application (Figure 3 and Figure 4), which predicts the probability of patients recovering their prefracture ADL status one year after the fracture, is accessible at www.calconic.com/calculator-widgets/hip-fracture-functional-recovery-prediction-tool/64903a84b4cd1f001eb92003?layouts=true accessed on 12 January 2024. It provides clinicians with a tool that can be used to estimate recovery chances and guide treatment planning.

The model stratifies patients into one of two tiers: high probability of regaining prefracture ability or low probability of regaining prefracture ability, as shown in Table 3. A predicted LHR less than 1.3 indicates a higher risk of not achieving baseline functional status at one year, suggesting the need for more intensive management. Conversely, an LHR of 1.3 or above justifies adherence to the standard treatment protocols.

- Scenario 1:

As shown in Figure 3, we demonstrated the model’s function using a case of a patient who had sustained an intertrochanteric fracture of femur and whose treatment team planned to treat them with cephalomedullary nailing. The patient had a pre-fracture BI of 100 and pre-fracture EQ-VAS of 100. After all parameters were input into the web application, the algorithm yielded an LHR of 4.42, indicating that the patient was likely to return to pre-fracture activities-of-daily-living status at one year following fragility hip fracture. Given these results, the caregiving physician could follow the standard surgical treatment protocol with a regular post-operative plan for this patient.

- Scenario 2:

As shown in Figure 4, we also demonstrated the model’s function using a case of a patient who had suffered a non-displaced femoral neck fracture and whose treatment team planned to treat them with conservative management due to an unstable cardiac condition. The patient had a pre-fracture BI of 60 and pre-fracture EQ-VAS of 60. After all parameters were input into the web application, the algorithm yielded an LHR of 0.0009, indicating that the patient was not likely to return to pre-fracture activities-of-daily-living status at one year following fragility hip fracture. Therefore, the caregiving physician should plan for more intensive management to maximize the chances of functional recovery. This plan may include a cardiologist consultation for close monitoring and urgently managing the heart condition and other active medical issues. Next, the physician should engage in full communication with the patient and family about the risks and benefits of all treatment options, including surgery, to allow for prompt clinical decision-making as soon as the patient’s condition was optimized and the patient was considered medically fit for interventions. The intensive care unit should also be booked in case it is needed preoperatively or postoperatively. Additional services including cardiac rehabilitation, personalized physical therapy program and home visits by a family practitioner may also be beneficial in this case to support the patient in regaining baseline functional status and to prevent both short- and long-term complications.

## 4. Discussion

Our study established clinical prediction criteria validated with three significant predictors: the prefracture BI score, EQ-VAS score, and chosen treatment approach. The model demonstrated strong discriminative capability and reliable calibration in forecasting patients’ recovery one year after a fragility hip fracture.

Hip-fracture rehabilitation typically follows a nonlinear pattern that is characterized by substantial early improvements within the first 2 months and a more gradual progression after that [22]. The likelihood of rehabilitation restoring prefracture function remains uncertain [23]. Nonetheless, the literature consistently supports the benefits of prompt rehabilitation, which significantly improves mobility, capacity for self-care, and independence in ADL [14], ultimately enhancing the quality of life for older patients with hip fractures [24]. Given that the recovery of ADLs can continue for up to a year [13], an intensive rehabilitation program during this period is strongly recommended, especially for those at higher risk.

Current models for prediction of functional recovery after hip fracture are scarce, with most tools focusing on the outcomes of hip-fracture fixation. For instance, models developed by Hsu et al. [25] and Phruetthiphat et al. [26] predict outcomes for specific fracture types and treatments. Hsu et al. developed a scoring system to predict treatment failure in elderly patients with intertrochanteric fracture treated only with a dynamic hip screw [25]. Phruetthiphat et al. also developed a scoring system for predicting functional outcomes in elderly patients with intertrochanteric fracture treated only with a cephalomedullary nailing system [26]. In contrast, Tanaka et al. proposed a model for predicting ADL outcomes up to 6 months after surgery [27]; however, this period does not cover the crucial long-term recovery phase.

Given that a year or more is often needed for recovery of physical function [13], we developed clinical prediction criteria capable of estimating the likelihood of patients regaining their prefracture functional status 1 year after the fracture. Therefore, compared to the available prediction tools, our model provides a prediction for longer-term regain of function in all types of fragility hip fractures (e.g., fracture of the femoral neck or intertrochanteric fracture) treated with all types of treatment options (e.g., conservative treatment, dynamic hip screw fixation, cephalomedullary nailing, multiple screw fixation, or arthroplasty) simulating real-world clinical practices for fragility hip-fracture care. This advancement offers a valuable tool to aid clinicians in planning and optimizing rehabilitation strategies for hip-fracture patients.

There were several prognostic variables that were already known to help predict patients’ ability to return to pre-fracture functional status at 1 year following a fragility hip fracture; such predictions could be used to advance knowledge of the long-term effects of fragility hip fractures and help create focused interventions to increase functional recovery in this group of patients [28,29]. For instance, baseline patient demographics, fracture-related factors, variables pertaining to the caregiving process, psychosocial factors, cognitive factors, and socio-economic factors were considered to have an impact on the patient’s physical recovery from the consequences of fragility hip fractures [29]. Our previous research explored prognostic factors for one-year functional recovery after fragility hip fractures, identifying three key predictors: a CCI of less than 5, surgical treatment, and a prefracture EQ-VAS score of 65 or above [21]. Age, CCI score, treatment type, prefracture BI score, and prefracture EQ-VAS score were initially selected as candidate predictors based on the literature suggesting their link to positive recovery outcomes. It has been proposed that advanced age and multiple chronic illnesses are important indicators of impaired physical function in patients with fragility hip fractures because older patients are likely to have more comorbidities and are more susceptible to adverse events after hospitalization due to impaired cognitive function, which limits their ability to help themselves, delaying rehabilitation and prolonging their length of stay [30]. By contrast, patients who are younger and have fewer comorbidities tend to have better functional recovery. Surgical treatment also allows patients to access early rehabilitation programs and facilitates training that can allow them to return to their previous level of activities of daily living [31]. Pre-fracture BI and pre-fracture EQ-VAS, which assess baseline physical function and overall health status, respectively, also provide crucial information about the initial level of independence and quality of life and thus determine the patient’s capacity to recover long-term physical function [21,32]. These factors offer insights into patients’ initial health status and treatment plans and enable healthcare providers, patients, and families to understand the expected outcomes [33]. Gender was also reported to be associated with functional recovery after geriatric hip fractures [34]. However, we did not find a difference in functional outcomes according to gender.

In a real-world situation, it was not possible to make individual predictions by taking each of the previously listed prognostic factors into account separately. By using statistical modeling methods to develop a clinical prediction algorithm, we were able to make use of multiple prognostic factors to categorize each patient’s likelihood of regaining preinjury activities-of-daily-living status as high or low. Our subsequent analysis adjusted the approach by treating the CCI as a continuous variable and applying the MFP method. This adjustment led to the exclusion of the CCI from the final model due to its nonsignificant impact on recovery. In contrast, the prefracture BI was linearly associated with patient outcomes and was included. Unique to our model is the inclusion of the prefracture EQ-VAS score, a factor overlooked by the developers of previous tools. Our analyses determined that this score is a significant predictor of recovery, and its inclusion in the model underscores the critical role of patients’ baseline health status.

The strength of this study lies in its comprehensive approach to predicting the long-term functional recovery of both surgically and conservatively treated patients. The model’s predictions are based on readily available admission data and can aid in making informed decisions about treatment and care strategies to maximize the patient’s chances of recovery. By emphasizing function, independence, and well-being, the model addresses the critical needs of older hip-fracture patients and supports identifying those at higher risk. Moreover, by accounting for the global increase in fragility hip fractures, our model offers valuable insights into managing this growing healthcare challenge.

Nonetheless, our study has certain limitations worth noting. First, it excludes variables such as postoperative complications that might affect perioperative outcomes and the probability of functional recovery following a hip fracture. We opted to focus on the predictive value of data available at admission and hence excluded posttreatment variables from our model. Second, the fact that the study’s sample is relatively small and sourced from a single center could potentially lead to the risk of model overfitting. This limitation underscores the need for external validation to confirm our model’s discriminative ability across broader populations. Finally, the accuracy of predictions is contingent upon the selected cutoff criterion for each variable and on how the return to prefracture functionality is defined.

## 5. Conclusions

Our predictive model offers significant discriminative ability and reliable calibration for predicting one-year recovery outcomes after a fragility hip fracture. The model provides clinicians with a valuable tool to aid in making individualized recovery prognoses at admission by categorizing the patient’s likelihood of regaining preinjury ADL status as high or low. Furthermore, this model can inform targeted counseling and facilitate decision-making regarding effective recovery strategies after a hip fracture, emphasizing the importance of further research and external validation to enhance the model’s applicability and generalizability.

## Figures and Tables

**Table 1:** Comparative analysis of demographic and clinical profiles across patient cohorts based on recovery of prefracture activities-of-daily-living status.

Patient Characteristics	Return to Prefracture Activities-of-Daily-Living Status (*n* = 324)	Did Not Return to Prefracture Activities-of-Daily-Living Status *(n* = 97)	Odds Ratio	95% CI	*p*-Value	auROC
Age (y), mean ± SD	77.33 ± 9.65	81.35 ± 8.39	0.95	[0.93–0.98]	<0.001	0.61 [0.55–0.67]
Female sex, *n* (%)	242 (74.69%)	76 (78.35%)	0.82	[0.47–1.41]	0.463	0.52 [0.47–0.57]
Body mass index (kg/m^2^), mean ± SD	22.68 ± 4.11	22.12 ± 4.02	1.03	[0.98–1.10]	0.24	0.54 [0.47–0.60]
Charlson comorbidity index score, *n* (%)						
–<5	178 (54.94%)	31 (31.96%)	2.6	[1.61–4.19]	<0.001	0.61 [0.56–0.67]
Type of fracture, *n* (%)						
–Femoral neck fracture	178 (54.94%)	46 (47.42%)	1.35	[0.86–2.13]	0.19	0.54 [0.48–0.59]
Treatment, *n* (%)						
–Conservative treatment	3 (0.93%)	14 (14.43%)				0.60 [0.53–0.66]
–Dynamic hip screw	21 (6.48%)	6 (6.19%)	16.33	[3.49–76.35]	<0.001	
–Cephalomedullary nailing	133 (41.05%)	40 (41.24%)	15.52	[4.25–56.71]	<0.001	
–Multiple screw fixation	14 (4.32%)	2 (2.06%)	32.67	[4.71–226.52]	<0.001	
–Arthroplasty	153 (47.22%)	35 (36.08%)	20.4	[5.56–74.85]	<0.001	
Prefracture BI, mean ± SD	96.7 ± 6.02	80.72 ± 17.79	1.16	[1.12–1.20]	<0.001	0.83 [0.79–0.88]
Prefracture EQ-VAS, mean ± SD	92.48 ± 7.75	79.18 ± 16.29	1.11	[1.08–1.14]	<0.001	0.76 [0.70–0.82]

A *p*-value < 0.05 indicates statistical significance. Abbreviations: auROC, area under the receiver operating characteristic curve; BI, Barthel index; CI, confidence interval; SD, standard deviation.

**Table 2:** Multivariable fractional polynomial logistic regression analysis for prognostication of recovery to prefracture activities-of-daily-living status after fragility hip fracture.

Predictors	Terms	df	Formula	Log Odds Ratio	95% CI	*p*-Value
Intercept				0.26	−1.25, 1.76	0.739
Age	Out	0	–	–	–	–
CCI	Out	0	–	–	–	–
Treatment	Linear	1				
–Dynamic hip screw				2.14	0.29, 3.98	0.02
–Cephalomedullary nailing				2.06	0.53, 3.6	0.01
–Multiple screw fixation				3.3	0.48, 6.12	0.02
–Arthroplasty				2.08	0.54, 3.62	0.01
Prefracture Barthel index	Linear	1	Prefracture Barthel index-100	0.12	0.08, 0.16	<0.001
Prefracture EQ-VAS	Linear	1	Prefracture EQ-VAS-90	0.04	0.001, 0.07	0.045

A *p*-value < 0.05 indicates statistical significance. Abbreviations: CCI, Charlson comorbidity index; CI, confidence interval; df, degrees of freedom; SD, standard deviation.

**Table 3:** Proportional representation of patients achieving prefracture activities-of-daily-living status one year after fragility hip fracture.

Categories	LHR to Return to Prefracture Activties-of-Daily-Living Status	Return to Prefracture Activities-of-Daily-Living Status		Did Not Return to Prefracture Activities-of-Daily-Living Status		LR+	95% CI of LR+	PPV	95% CI of PPV	*p*-Value
		*n*	%	*n*	%					
Low	<1.3	68	21	79	81.4	0.26	0.20 0.32	46.3%	38.0% 54.7%	<0.001
High	≥1.3	256	79	18	18.6	4.26	2.80 6.48	93.4%	89.8% 96.1%	<0.001

A *p*-value < 0.05 indicates statistical significance. Abbreviations: CI, confidence interval; LHR, likelihood ratio; LR+, likelihood ratio of positive; PPV, positive predictive value.

## Data Availability

Data will be provided from corresponding authors upon reasonable request.

## References

[1] Kanis J.A., McCloskey E.V., Johansson H., Cooper C., Rizzoli R., Reginster J.Y. (2013). European guidance for the diagnosis and management of osteoporosis in postmenopausal women. Osteoporos. Int..

[2] Chapurlat R.D., Bauer D.C., Nevitt M., Stone K., Cummings S.R. (2003). Incidence and risk factors for a second hip fracture in elderly women. The Study of Osteoporotic Fractures. Osteoporos. Int..

[3] Center J.R., Nguyen T.V., Schneider D., Sambrook P.N., Eisman J.A. (1999). Mortality after all major types of osteoporotic fracture in men and women: An observational study. Lancet.

[4] Peek-Asa C., Zwerling C. (2003). Role of environmental interventions in injury control and prevention. Epidemiol. Rev..

[5] Vaseenon T., Luevitoonvechkij S., Wongtriratanachai P., Rojanasthien S. (2010). Long-term mortality after osteoporotic hip fracture in Chiang Mai, Thailand. J. Clin. Densitom..

[6] Vochteloo A.J., Moerman S., Tuinebreijer W.E., Maier A.B., de Vries M.R., Bloem R.M., Nelissen R.G., Pilot P. (2013). More than half of hip fracture patients do not regain mobility in the first postoperative year. Geriatr. Gerontol. Int..

[7] Colón-Emeric C., Kuchibhatla M., Pieper C., Hawkes W., Fredman L., Magaziner J., Zimmerman S., Lyles K.W. (2003). The contribution of hip fracture to risk of subsequent fractures: Data from two longitudinal studies. Osteoporos. Int..

[8] Aubrun F. (2011). Hip fracture surgery in the elderly patient: Epidemiological data and risk factors. Ann. Fr. Anesth. Reanim..

[9] Gullberg B., Johnell O., Kanis J.A. (1997). World-wide projections for hip fracture. Osteoporos. Int..

[10] Wongtriratanachai P., Luevitoonvechkij S., Songpatanasilp T., Sribunditkul S., Leerapun T., Phadungkiat S., Rojanasthien S. (2013). Increasing incidence of hip fracture in Chiang Mai, Thailand. J. Clin. Densitom..

[11] Sukchokpanich P., Anusitviwat C., Jarusriwanna A., Kitcharanant N., Unnanuntana A. (2023). Quality of Life and Depression Status of Caregivers of Patients with Femoral Neck or Intertrochanteric Femoral Fractures during the First Year after Fracture Treatment. Orthop. Surg..

[12] Scaf-Klomp W., van Sonderen E., Sanderman R., Ormel J., Kempen G.I. (2001). Recovery of physical function after limb injuries in independent older people living at home. Age Ageing.

[13] Magaziner J., Hawkes W., Hebel J.R., Zimmerman S.I., Fox K.M., Dolan M., Felsenthal G., Kenzora J. (2000). Recovery from hip fracture in eight areas of function. J. Gerontol. A Biol. Sci. Med. Sci..

[14] Huusko T.M., Karppi P., Avikainen V., Kautiainen H., Sulkava R. (2002). Intensive geriatric rehabilitation of hip fracture patients: A randomized, controlled trial. Acta Orthop. Scand..

[15] Wawruch M., Krcmery S., Bozekova L., Wsolova L., Lassan S., Slobodova Z., Kriska M. (2004). Factors influencing prognosis of pneumonia in elderly patients. Aging Clin. Exp. Res..

[16] Mahaisavariya C., Vanitcharoenkul E., Kitcharanant N., Chotiyarnwong P., Unnanuntana A. (2023). Exploring the osteoporosis treatment gap after fragility hip fracture at a Tertiary University Medical Center in Thailand. BMC Geriatr..

[17] Sinoff G., Ore L. (1997). The Barthel activities of daily living index: Self-reporting versus actual performance in the old-old (> or = 75 years). J. Am. Geriatr. Soc..

[18] Unnanuntana A., Jarusriwanna A., Nepal S. (2018). Validity and responsiveness of Barthel index for measuring functional recovery after hemiarthroplasty for femoral neck fracture. Arch. Orthop. Trauma Surg..

[19] Della Pietra G.L., Savio K., Oddone E., Reggiani M., Monaco F., Leone M.A. (2011). Validity and reliability of the Barthel index administered by telephone. Stroke.

[20] Kim S., Won C.W., Kim B.S., Kim S., Yoo J., Byun S., Jang H.C., Cho B.L., Son S.J., Lee J.H. (2018). EuroQol Visual Analogue Scale (EQ-VAS) as a Predicting Tool for Frailty in Older Korean Adults: The Korean Frailty an Aging Cohort Study (KFACS). J. Nutr. Health Aging.

[21] Kitcharanant N., Atthakomol P., Khorana J., Phinyo P., Unnanuntana A. (2024). Prognostic Factors for Functional Recovery at 1-Year Following Fragility Hip Fractures. Clin. Orthop. Surg..

[22] Mathew R.O., Hsu W.H., Young Y. (2013). Effect of comorbidity on functional recovery after hip fracture in the elderly. Am. J. Phys. Med. Rehabil..

[23] Amarilla-Donoso F.J., Roncero-Martin R., Lavado-Garcia J.M., Toribio-Felipe R., Moran-Garcia J.M., Lopez-Espuela F. (2020). Quality of life after hip fracture: A 12-month prospective study. PeerJ.

[24] Shyu Y.I., Chen M.C., Liang J., Lu J.F., Wu C.C., Su J.Y. (2004). Changes in quality of life among elderly patients with hip fracture in Taiwan. Osteoporos. Int..

[25] Hsu C.E., Huang K.C., Lin T.C., Tong K.M., Lee M.H., Chiu Y.C. (2016). Integrated risk scoring model for predicting dynamic hip screw treatment outcome of intertrochanteric fracture. Injury.

[26] Phruetthiphat O.A., Pinijprapa P., Satravaha Y., Kitcharanant N., Pongchaiyakul C. (2022). An innovative scoring system for predicting an excellent Harris hip score after proximal femoral nail anti-rotation in elderly patients with intertrochanteric fracture. Sci. Rep..

[27] Tanaka R., Umehara T., Fujimura T., Ozawa J. (2016). Clinical Prediction Rule for Declines in Activities of Daily Living at 6 Months After Surgery for Hip Fracture Repair. Arch. Phys. Med. Rehabil..

[28] Sheehan K.J., Williamson L., Alexander J., Filliter C., Sobolev B., Guy P., Bearne L.M., Sackley C. (2018). Prognostic factors of functional outcome after hip fracture surgery: A systematic review. Age Ageing.

[29] Lim K.K., Matchar D.B., Chong J.L., Yeo W., Howe T.S., Koh J.S.B. (2019). Pre-discharge prognostic factors of physical function among older adults with hip fracture surgery: A systematic review. Osteoporos. Int..

[30] Kim J.L., Jung J.S., Kim S.J. (2016). Prediction of Ambulatory Status After Hip Fracture Surgery in Patients Over 60 Years Old. Ann. Rehabil. Med..

[31] Malhotra R., Huq S.S., Chong M., Murphy D., Daruwalla Z.J. (2021). Outcomes in Nonagenarians with Hip Fractures Treated Conservatively and Surgically. Malays. Orthop. J..

[32] Mayoral A.P., Ibarz E., Gracia L., Mateo J., Herrera A. (2019). The use of Barthel index for the assessment of the functional recovery after osteoporotic hip fracture: One year follow-up. PLoS ONE.

[33] Bliemel C., Sielski R., Doering B., Dodel R., Balzer-Geldsetzer M., Ruchholtz S., Buecking B. (2016). Pre-fracture quality of life predicts 1-year survival in elderly patients with hip fracture-development of a new scoring system. Osteoporos. Int..

[34] Trovarelli G., Crimì A., Mori F., De Martini N.F., Cerchiaro M.C., Ruggieri P. (2023). Proximal femur fractures in elderly patients: Gender-related differences in survival and functional outcomes. J. Sex-Gend.-Specif. Med..

